# Holistic Therapy in a Patient with Necrotic Ulcer Caused by the Bite of Brazilian Wandering Spider: A Case Report of Challenging Treatment with Combined Therapies

**DOI:** 10.3390/jcm15020693

**Published:** 2026-01-15

**Authors:** Anna Hepa-Banasik, Magdalena Szatan, Anna Słaboń, Jarosław Łach, Artur Wielgórecki, Katarzyna Czerny-Bednarczyk, Wojciech Łabuś

**Affiliations:** 1 Stanislaw Sakiel Burn Treatment Centre in Siemianowice Śląskie, Jana Pawła II Street 2, 41-100 Siemianowice Śląskie, Polandartur.wielgorecki@clo.com.pl (A.W.); wojciech.labus@clo.com.pl (W.Ł.); 2 Doctoral School of the Medical University of Silesia in Katowice, Faculty of Health Sciences in Bytom, Medical University of Silesia in Katowice, 41-902 Bytom, Poland

**Keywords:** chronic wound, spider bite, acellular dermal matrix, split thickness skin graft, microperfusion

## Abstract

Hard-to-heal wounds remain a significant challenge for healthcare professionals, particularly in aging populations. Although most chronic wounds are associated with diabetes or chronic venous insufficiency, rare etiologies should also be considered. One such cause is envenomation by *Phoneutria* spp. (native to South America, rare in Europe). Their venom contains potent neurotoxins. While systemic manifestations are more commonly reported, localized necrotic skin lesions may also occur. This case report presents a rare chronic wound following a suspected *Phoneutria* spider bite and highlights the importance of an individualized, multimodal treatment approach. A 61-year-old male patient with a progressive thigh wound following a spider bite sustained during work. Despite initial self-treatment and pharmacotherapy the wound deteriorated. The patient was admitted to the authors’ facility, where surgical treatment included necrosectomy and a sandwich graft using an acellular dermal matrix combined with a split-thickness skin graft. Adjunctive therapies included negative pressure wound therapy and hyperbaric oxygen therapy. After discharge, outpatient wound care was continued. Treatment was monitored with photographic documentation and serial microperfusion measurements. Complete wound closure was achieved after 4 months of specialized therapy. Management of chronic wounds requires a multidisciplinary and individualized approach with surgical intervention, advanced wound care and specialized outpatient follow-up.

## 1. Introduction

Chronic wounds represent a growing challenge for healthcare systems both in Poland and worldwide [1]. A chronic wound is one that fails to progress through the normal phases of healing in a timely and orderly manner, often becoming stalled in the inflammatory phase. Typically, a wound is classified as chronic if it shows no signs of healing after more than six weeks despite appropriate treatment [2]. This situation may be due to the long-observed phenomenon of population aging. In this context, studies have shown that the average age of patients with chronic wounds is approximately 70–80 years [3]. The increasing proportion of post-working-age individuals contributes to the growing burden of chronic wounds. It has been demonstrated that the majority of chronic wounds are lower limb ulcers, occurring in 1.51 per 1000 people [1,2,3,4,5]. Furthermore, the most common underlying causes include diabetic ulcers (41.5%), venous insufficiency (24.5%), and pressure injuries (13.2%) [4]. The frequent occurrence of wounds with the same etiology has led to the development of numerous scientific studies presenting effective treatment strategies.

It should also be noted that the prolonged healing time of chronic wounds often generates high treatment costs. In Europe, the cost of treating diabetic foot ulcers alone is estimated at 4–6 billion euros annually [5].

Wounds caused by bites from spiders of the Ctenidae family constitute a small fraction of chronic wound cases in Europe. However, in South American countries such as Brazil, around 4600 bites are reported annually [6]. Most of these cases have a mild course, with only about 0.5% progressing to severe poisoning, primarily in children under the age of 10 and elderly individuals. In Argentina, at least 150 cases of *Phoneutria* bites are recorded annually, mainly in the northeastern region of the country [7]. The main contributing factor is that the venom contains a potent neurotoxin, PhTx3 (*Phoneutria* toxin 3). Since 1903, 15 deaths in Brazil have been attributed to *Phoneutria* bites, though only two cases have sufficient evidence to confirm a direct causal relationship. In Europe, *Phoneutria* bites are extremely rare and usually associated with the import of fruits, particularly bananas [8]. Cases such as the one reported in Poland are exceptional and of significant clinical interest. The name of this spider comes from its characteristic wandering behavior. The Brazilian wandering spider does not spin webs but actively roams in search of food. Belonging to the Ctenidae family, it is also known as the “banana spider” due to its presence on banana plants, often hiding within the fruit clusters [6]. Its nomadic lifestyle is often the reason for accidental transportation to other parts of the world. The spider inhabits tropical forests of South and Central America, especially Brazil, Colombia, Venezuela, and Ecuador. It measures between 3 and 5 cm in body length and has a brown or grayish color with darker stripes on its legs. The Brazilian wandering spider produces a potent neurotoxin (*Phoneutria* toxin, Tx2-6) in its venom glands, which affects the nervous system. The most common clinical manifestation of a bite is immediate localized pain, reported in over 90% of cases. The pain is often severe and radiating [6]. Additional local symptoms include edema without induration, erythema, and paresthesia [6,9]. Life-threatening symptoms include pulmonary edema and shock resulting from envenomation, especially in elderly individuals and children [6,9]. In a 2023 study by Bucaretchi et al., a retrospective cohort case series of patients admitted to an emergency department after *Phoneutria* bites (phoneutrism) was analyzed. The study examined patients’ reported pain using the Numeric Pain Rating Scale (NPRS 0–10) and recorded the analgesics administered. Based on the analysis, pain management pathways tailored to the declared pain levels were proposed [9,10].

The primary aim of presenting this case was to provide clinicians with practical guidance on the management of highly complex wounds resulting from rare toxic etiologies. Toxic epidermal necrolysis of the skin following a spider bite represents an exceptionally uncommon clinical condition, with which most medical professionals are unlikely to gain direct experience during their careers. Moreover, the limited number of reported cases in the literature results in a lack of clearly described therapeutic pathways. As a specialized center routinely involved in advanced wound management, we sought to share a structured and multidisciplinary treatment protocol that led to successful wound healing in this patient. By documenting our diagnostic approach and stepwise therapeutic strategy, we aim to contribute to the existing literature and offer a potential reference framework for clinicians faced with similarly challenging and rare wound presentations. As a hospital with a tissue bank on site and many years of experience in treating the most severe burns, toxic epidermal necrolysis (TEN), and chronic wounds, we wanted to show how the most advanced medical technologies can be used to cure a patient with a very rare type of wound.

## 2. Materials and Methods

The medical procedures used in this study were performed in accordance with the ethical standards of the Declaration of Helsinki (1964, last amended in 2008).

All the activities performed were recognized and formally approved forms of treatment. In addition, all the activities performed were aimed at improving the patient’s health, and in certain cases, constituted a desperate attempt to save his health and life. According to Polish law, retrospective observational studies, including case reports, do not require approval from a bioethics committee.

The patient gave informed consent to hospitalization and to the publication of medical data and images.

### Case Presentation

**Figure 1:**
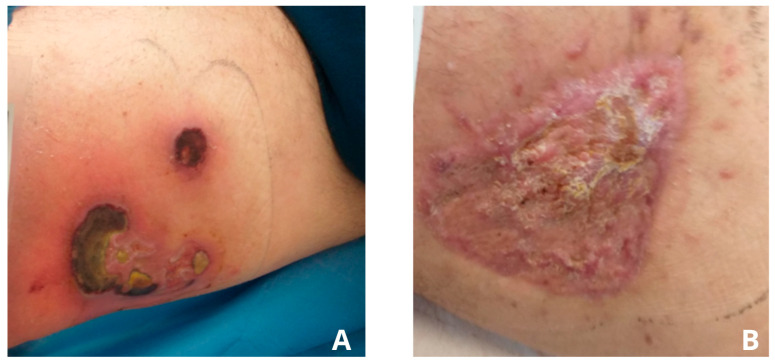
(**A**)—Photo taken by the patient one day prior to hospital admission for the planned surgical demarcation of necrotic tissue (19 August 2024); (**B**)—Photo taken at the Ambulatory Nursing Care (ANC) clinic during the follow-up visit (5 November 2024).

The patient, a 61-year-old male, presented on 5 August 2024, to the General Surgery Outpatient Clinic at the Stanisław Sakiel Burn Treatment Center (CLO) due to a wound located on the medial aspect of the right thigh, which had developed approximately two months prior to the visit (Figure 1). During the medical interview, it was determined that the wound originated at the patient’s workplace while performing routine cargo unloading duties. The patient works as a fruit vendor, including handling bananas. While unloading one of the boxes, he was bitten by a Brazilian wandering spider (*Phoneutria* spp.). Initially, the patient attempted self-treatment. Unfortunately, in the days following the bite, the wound condition deteriorated. Due to the worsening condition, the man sought help at a local surgical outpatient clinic near his residence and later visited a private surgical practice. The patient also had comorbidities, including diabetes and arterial hypertension.

## 3. Results

During treatment at the local clinic, the patient was prescribed clindamycin and amoxicillin-clavulanic acid (Amoksiklav). Additionally, topical povidone-iodine ointment and a silver-impregnated dressing were applied. During physical examination at the surgical outpatient clinic at the Burn Treatment Center, an inflammatory area approximately 15 cm^2^ in size was observed, with three areas of dry skin necrosis, each about 2 cm in diameter. A moist dressing with sodium hypochlorite was applied, and the patient was instructed to continue wound hygiene at home and apply dressings containing sodium polyacrylate activated with Ringer’s solution and polyhexamethylene biguanide.

Despite ongoing treatment, about 60 days after the spider bite, the condition of the wound continued to deteriorate. Necrolytic skin damage developed. A follow-up examination on 19 August 2024, revealed a wound approximately 15 cm^2^ in size, featuring deep ulcerations with necrotic tissue and loss of subcutaneous tissue. The surrounding skin showed marked inflammation (Figure 1A).

An urgent hospital admission was arranged to the Institute of Chronic Wounds at the CLO (ICW). The hospitalization process included two surgical procedures, a 15-day inpatient stay, 37 hyperbaric oxygen therapy (HBOT) sessions (2.5 ATA for 1 h. Seven sessions during hospitalization and 30 sessions at ANC), and 16 outpatient nursing care visits following discharge in period of 7 days.

During the stay at ICW the first surgical procedure, was performed on 22 August 2024. During the surgery the wound was debrided, including the excision of necrotic adipose tissue and venesection of a necrotic segment of the great saphenous vein down to the muscle fascia. The wound was secured using a negative pressure wound therapy (NPWT) system set to −120 mmHg, continuous for 6 days.

During the second surgery performed on 28 August 2024 surgical debridement of necrotic tissue within the wound was performed. An acellular dermal matrix (ADM), meshed at a 1:2 ratio, was applied directly to the debrided wound surface, followed by a meshed split-thickness skin graft (STSG) harvested from the left thigh at a 1:1.5 ratio. The wound was then covered with a paraffin dressing and gauze impregnated with polyhexamethylene biguanide (PHMB). Following hospital discharge, approximately 80% of the graft underwent lysis.

**Figure 2:**
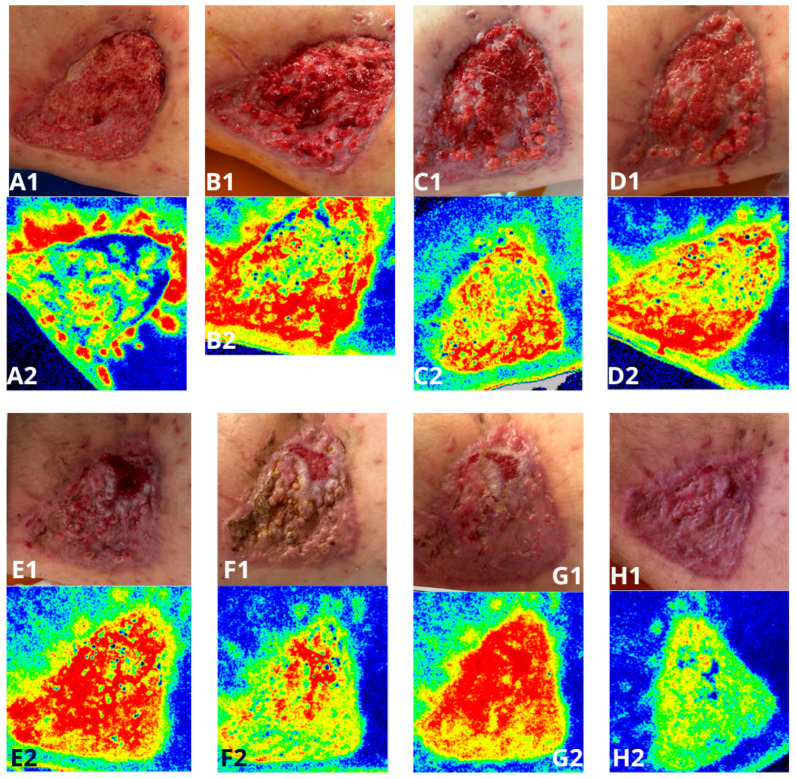
Photographs and microperfusion measurements of a chronic wound located on the medial aspect of the right thigh, taken during follow-up visits at the Ambulatory Nursing Care Clinic. (**A1**,**A2**)—Photograph and microperfusion measurement taken on 12 September 2024; (**B1**,**B2**)—Photograph and microperfusion measurement taken on 18 September 2024; (**C1**,**C2**)—Photograph and microperfusion measurement taken on 26 September 2024; (**D1**,**D2**)—Photograph and microperfusion measurement taken on 30 September 2024; (**E1**,**E2**)—Photograph and microperfusion measurement taken on 17 October 2024; (**F1**,**F2**)—Photograph and microperfusion measurement taken on 21 October 2024; (**G1**,**G2**)—Photograph and microperfusion measurement taken on 24 October 2024; (**H1**,**H2**)—Photograph and microperfusion measurement taken on 29 October 2024.

Further treatment continued through weekly visits at the Ambulatory Nursing Care clinic located within the General Surgery Outpatient Clinic at the Burn Treatment Center in Siemianowice Śląskie. During each visit, mechanical debridement of biofilm was performed. The wound surface was covered with serosanguinous exudate of a green-brown coloration, and a strong, foul odor was noted upon dressing removal. Despite these symptoms, the patient did not exhibit clinical signs of local or systemic infection. In the initial phase of treatment, each wound cleansing was followed by the application of a collagen dressing (containing type I and III collagen) with an extracellular matrix (ECM) structure, a gel containing hyaluronic acid and amino acids, and a lipidocolloid (TLC) dressing impregnated with silver. The primary dressing was covered with a secondary dressing capable of absorbing high levels of exudate. After four weeks of this therapy, the gel formulation was replaced with a powdered product containing hyaluronic acid and amino acids. The other types and sequence of dressings remained unchanged. Visits continued until full wound closure was achieved. Throughout the treatment process, microperfusion measurements (Figure 2) were performed using speckle laser imaging (Perimed AB, Järfälla, Sweden), along with photographic documentation to assess wound surface area. It is a technique for imaging blood flow in tissues (microperfusion), used primarily in the assessment of wound healing.

**Figure 3:**
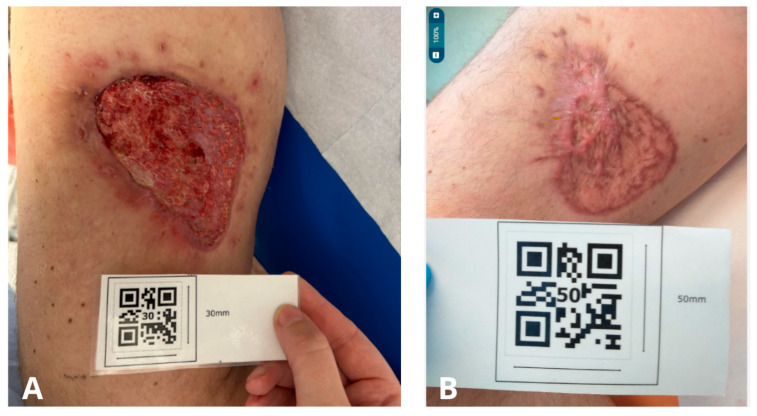
(**A**)—Photo taken by the patient one day prior to hospital admission for the planned surgical demarcation of necrotic tissue (12 September 2024); (**B**)—Photo taken at the General Surgery Clinic during the last follow-up visit (14 January 2025).

After discharge from the Institute for the Treatment of Chronic Wounds, the patient attended 16 visits at the Ambulatory Nursing Care Unit, during which complete wound healing was achieved (Figure 1B). Follow-up visits were conducted on a weekly basis. Figure 3A presents the wound at the time of hospital discharge, with the recommendation for continued outpatient care, whereas Figure 3B shows the scar following complete wound closure. Based on planimetric assessments performed during ambulatory care, the wound surface area at the initiation of outpatient treatment measured 43.8 cm^2^. After complete healing, the resulting scar covered an area of 26.6 cm^2^, corresponding to 100% wound closure.

## 4. Discussion

**Figure 4:**
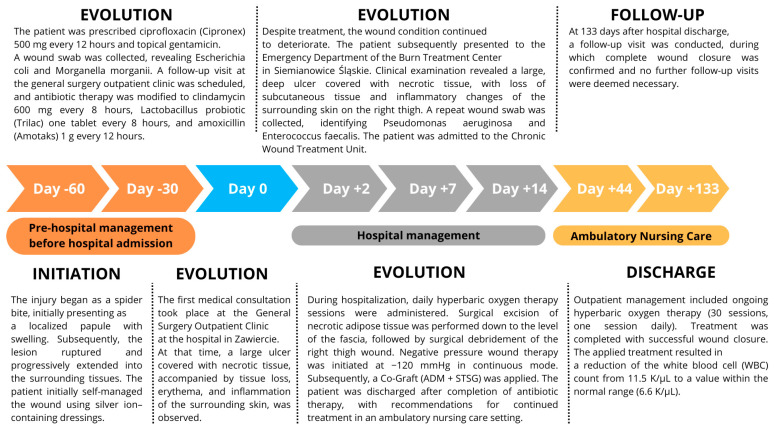
Timeline of clinical events.

The treatment of chronic wounds continues to be a major challenge for both medical personnel and healthcare systems. A significant portion of these cases involves wounds of diabetic and chronic venous insufficiency etiology. This trend is likely linked to the aging population in Poland and Europe, as well as changes in lifestyle and dietary habits [3,5,11,12]. Graves et al., in their study, estimated the costs associated with treating patients with chronic wounds in Singapore’s multiethnic population. The study included a group of 3.49 million Singaporean citizens and permanent residents living in the city in 2017. In that year, 16,752 cases of chronic wounds were identified (598 venous ulcers, 2206 arterial ulcers, 6680 diabetic wounds, and 7268 pressure injuries). The annual treatment costs were estimated at 350 million USD. The largest portion of the cost came from hospital stays—40% of all costs (139 million USD), while outpatient visits accounted for 10% (37 million USD). The study emphasized that chronic wounds pose a significant burden on healthcare systems. Implementing prevention pro-grams and patient education could significantly reduce costs and improve patients’ quality of life [12]. However, aside from these “most typical” chronic wound cases, there are wounds of much rarer etiologies. One such case is described in this paper. Each case requires an individualized approach by medical personnel. Based on a thorough patient history, the most optimal treatment protocol should be chosen. In addition to individual factors such as age, comorbidities, and medications, healthcare professionals must also consider the patient’s financial capacity, as treatment may last several to even over a dozen months. In the case described, the wound was characterized by heavy exudation, suggesting a strong inflammatory response to the spider venom and secondary necrotic processes. The green-brown color and strong odor could have resulted from protein breakdown, even in the absence of an active bacterial infection. The improper combination of iodine and silver ions may have delayed the healing process. The application of negative pressure wound therapy during the first surgical procedure likely contributed to appropriate wound bed preparation, thereby increasing the probability of graft integration during the subsequent intervention. It may be hypothesized that the observed adverse outcome, namely approximately 80% graft lysis, could have been associated with the persistent local effects of spider venom, specifically PhTx3 (*Phoneutria* toxin 3), retained within the tissues. The prolonged presence of the toxin may have contributed to an extended inflammatory response, thereby negatively affecting the graft take. Advanced chronic wound care methods offer effective and modern approaches to improve healing outcomes. In an article by Brown et al., special attention was given to the importance of a moist wound environment and the role of antibacterial dressings in preventing infection [10]. That publication presented the treatment of a 60-year-old man following a spider bite. Total wound healing time was 73 days, and closure was achieved through modern treatment techniques and regular follow-ups (Figure 4). Therapies such as NPWT (Negative Pressure Wound Therapy) and HBOT (Hyperbaric Oxygen Therapy) can significantly accelerate healing and improve quality of life for patients with difficult-to-treat wounds [13,14,15,16,17,18]. As indicated by Monami et al., supportive therapies like HBOT can actively promote wound healing and reduce healing time in patients with diabetic foot ulcers. HBOT and PRP/F (Platelet-Rich Plasma/ Fibrin) have also been shown to reduce the risk of major amputation [18]. Another method that may accelerate wound closure is the use of split-thickness skin grafts (STSG) and acellular dermal matrices (ADM). These techniques can be used in both chronic wounds and those resulting from surgical procedures. ADM and STSG may contribute to faster epithelialization and wound closure. However, the wound bed must be well-prepared (free of signs of infection) for the graft to successfully integrate. Numerous solutions are available on the market to maintain proper moisture in the wound and inhibit biofilm formation. The use of porcine type I and III collagen dressings and medical products containing sodium hyaluronate, amino acids responsible for collagen production, and silver ions contributed to tissue regeneration in this case. However, the treatment required a prolonged period and frequent dressing changes. Due to the rare etiology of the described wound, it was decided to conduct regular microperfusion measurements (Figure 2). The first measurement was taken in the Wound Treatment Unit at the Burn Treatment Center, with follow-up measurements conducted in outpatient nursing care (ANC). Many scientific publications highlight the benefits of using microperfusion monitoring in wound treatment [19,20,21,22,23]. This method allows for early detection of ischemic areas during hospitalization.

Several recent publications have described the management of wounds resulting from spider envenomation, highlighting both conservative and advanced therapeutic approaches [23,24,25]. Case reports and clinical reviews emphasize the importance of early wound assessment, appropriate surgical debridement, and careful monitoring of tissue viability to prevent progression of necrosis [23,26]. Among the reported treatment strategies, the use of negative pressure wound therapy (NPWT) has been described as a valuable adjunct in managing complex necrotic wounds following spider bites, promoting wound bed preparation and facilitating subsequent reconstruction [24,27]. Advanced wound care modalities, including collagen-based matrices and staged reconstructive techniques, have been successfully applied in cases complicated by extensive tissue loss [25,28]. In line with these reports, our case describes a 61-year-old male patient in whom successful wound closure was achieved following a suspected Brazilian wandering spider (*Phoneutria* spp.) bite, using a multimodal treatment approach that included surgical intervention and advanced wound therapies.

Spider venoms are complex mixtures of biologically active compounds, including neurotoxic peptides, enzymes, and pro-inflammatory mediators, which exert diverse effects on human tissues. In the case of spiders from the genus *Phoneutria*, venom activity is dominated by neurotoxins targeting voltage-gated sodium and calcium channels, leading to intense pain, neurogenic inflammation, and local vascular disturbances. Although *Phoneutria* venom is not primarily classified as necrotic, secondary tissue damage may occur as a consequence of severe inflammatory responses, microvascular dysfunction, and ischemia at the bite site. These processes can contribute to progressive tissue breakdown and, in rare cases, necrosis, particularly when diagnosis or intervention is delayed [29,30,31,32].

This study has several limitations that should be acknowledged. First, the report describes a single patient; however, cases of toxic skin necrolysis following spider envenomation are extremely rare, which limits the possibility of recruiting a larger cohort. Second, multiple therapeutic modalities were applied simultaneously during the treatment process, making it impossible to isolate and evaluate the individual effect of each intervention. Additionally, the duration of follow-up was limited, which may restrict the assessment of long-term outcomes. Finally, data collection was not initially planned in a structured or prospective manner, as the decision to document and publish this case was made during the course of therapy rather than prior to treatment initiation.

## 5. Conclusions

Chronic wound treatment requires a comprehensive approach from healthcare providers. The implementation of a multidirectional treatment model can contribute to faster wound closure. Key aspects of managing wounds with necrotic skin damage include access to specialized care, surgical wound debridement in a controlled hospital setting, and the use of advanced dressings and substances that support tissue regeneration.

## Data Availability

The original contributions presented in this study are included in the article.

## Abbreviations

The following abbreviations are used in this manuscript:

CLO	Stanislaw Sakiel Burn Treatment Center in Siemianowice Śląskie
ADM	Acellular Dermal Matrix
ANC	Ambulatory Nursing Care
ECM	Extracellular Matrix
HBOT	Hyperbaric Oxygen Therapy
ICW	Institute of Chronic Wounds
NPWT	Negative Pressure Wound Therapy
NPRS	Numeric Pain Rating Scale
PRP/F	Platelet-Rich Plasma/Fibrin
STSG	Split-Thickness Skin Graft
TLC	Technology Lipido-Colloid (Dressing Technology)
